# Human Neutrophil Elastase Induce Interleukin-10 Expression in Peripheral Blood Mononuclear Cells through Protein Kinase C Theta/Delta and Phospholipase Pathways

**DOI:** 10.22074/cellj.2016.3841

**Published:** 2016-01-17

**Authors:** Jin Kawata, Rui Yamaguchi, Takatoshi Yamamoto, Yasuji Ishimaru, Arisa Sakamoto, Manabu Aoki, Masafumi Kitano, Misako Umehashi, Eiji Hirose, Yasuo Yamaguchi

**Affiliations:** Graduate School of Medical Science, Kumamoto Health Science University, Kumamoto, Japan

**Keywords:** Interleukin-10, PBMC, Protein kinase C

## Abstract

**Objective:**

Neutrophils have an important role in the rapid innate immune response, and the release or active secretion of elastase from neutrophils is linked to various inflammatory responses. Purpose of this study was to determine how the human neutrophil elastase affects the interleukin-10 (IL-10) response in peripheral blood mononuclear cells (PBMC).

**Materials and Methods:**

In this prospective study, changes in IL-10 messenger RNA (mRNA) and protein expression levels in monocytes derived from human PBMCs were investigated after stimulation with human neutrophil elastase (HNE). A set of inhibitors was used for examining the pathways for IL-10 production induced by HNE.

**Results:**

Reverse transcription polymerase chain reaction (RT-PCR) showed that stimulation with HNE upregulated *IL-10* mRNA expression by monocytes, while the enzyme-linked immunosorbent assay (ELISA) revealed an increase of IL-10 protein level in the culture medium. A phospholipase C inhibitor (U73122) partially blunt- ed the induction of *IL-10* mRNA expression by HNE, while *IL-10* mRNA expression was significantly reduced by a protein kinase C (PKC) inhibitor (Rottlerin). A calcium chelator (3,4,5-trimethoxybenzoic acid 8-(diethylamino)octyl ester: TMB-8) inhibited the response of *IL-10* mRNA to stimulation by HNE. In addition, pretreatment with a broad-spectrum PKC inhibitor (Ro-318425) partly blocked the response to HNE. Finally, an inhibitor of PKC theta/delta abolished the increased level of *IL-10* mRNA expression.

**Conclusion:**

These results indicate that HNE mainly upregulates *IL-10* mRNA ex- pression and protein production in moncytes via a novel PKC theta/delta, although partially via the conventional PKC pathway.

## Introduction

Neutrophils are an important part of the rapid innate immune response that accompanies acute inflammation. Neutrophils release the contents of various granules while migrating to sites of inflammation, and these granule proteins play a central role in the early inflammatory response. The serine proteases cathepsin G, human leukocyte elastase, and proteinase 3 are abundant in neutrophil granules (1). These proteases regulate inflammatory processes by activating specific receptors and modulating the production of various cytokines (2). Human neutrophil elastase (HNE) is a 29 kDa serine endoprotease from the proteinase S1 family that forms a single 238-amino acid peptide chain with four disulfide bonds. Elastase released from activated neutrophils can cause tissue destruction (3-5), and is an important mediator of inflammatory tissue damage that is involved in the degradation of extracellular matrix components such as elastin, fibronectin, proteoglycan, and collagen (6). Serine proteases play various important physiological roles via G protein-coupled protease-activated receptors (PARs). PAR2 is a trypsin-activated member of the family of Gprotein- coupled PARs (7).

Inflammatory response-associated neutrophil proteinases may influence cell signaling by targeting PAR-2. It was reported that trypsin and the PAR-2 synthetic peptide agonist Ser-Leu- Ile-Gly-Arg-Leu (SLIGRL) induce Ca^2+^ mobilization, a transient increase of the inositol 1, 4, 5-trisphosphate (IP3) level, and translocation of protein kinase C (PKC) (8). Binding of HNE to PAR-2 increases the cytosolic calcium concentration and subsequently activates PKC, resulting in the secretion of mucin 5AC (MUC5AC) by airway epithelial cells (9). It was recently suggested that PAR-2 is involved in proinflammatory immune responses. Monocytes are well known to produce a range of pro-inflammatory and anti-inflammatory mediators. After activation by PAR-2, monocytes produce interleukin (IL)-6, IL-8, and IL-1beta, suggesting that PAR-2 may have a pro-inflammatory role (10). However, it is also possible that monocytes activated by PAR-2 produce IL-10 and thus have an anti-inflammatory effect. Accordingly, the present study investigated the possibility that PAR-2 participates in the regulation of antiinflammatory responses through the induction of IL-10 production by monocytes stimulated with HNE. The PKC family of serine/threonine kinases is composed of 10 isoforms that are divided into three classes, which are conventional (alpha, beta1, beta 2, and gamma), novel (delta, epsilon, theta, and eta) and atypical (xi and zeta) isoforms. We also examined the roles of these PKC isoforms in IL-10 production by monocytes.

## Materials and Methods

### Ethical considerations for prospective study

All human materials such as the peripheral blood cells used in this study were collected from male (n=15) and female (n=15) non-smoking healthy volunteers aged 26-33 after their informed consent was obtained. The protocol of this study was approved by Institutional Review Board of Kumamoto Health Science University, and the study was conducted in accordance with the Declaration of Helsinki.

### Reagents

HNE with 81 U/mg of activity was purchased from SERVA Electrophoresis (Heidelberg, Germany). 3,4,5-trimethoxybenzoic acid 8-(diethylamino) octyl ester (TMB-8, Sigma-Aldrich, Canada), A23187 (Santa Cruz Biotechnology, USA), Rottlerin Tumor necrosis factor-alpha (TNF-α)protease inhibitor I (TAPI-1), U73122, R59022 (Merck Millipore, USA), and pyrrolidinedithiocarbamate (PDTC, BioVision, USA) were employed to investigate the intracellular signal transduction pathways involved in IL-10 mRNA expression. The actions of these reagents are summarized in table 1.

In addition, PKC inhibitors including Ro- 318425 (Merck Millipore), Go 6976, Go 6983, and CGP 41251 (Tocris Bioscience, UK), as well as a PKC theta/delta inhibitor (Merck Millipore) were utilized to investigate the roles of PKC isoforms in IL-10 production. The PKC isoform-specific inhibition profile of these reagents is summarized in table 2.

**Table 1:** Functional characteristics of chemical agents used


Chemical agents	Functions

Rottlerin	Protein kinase C inhibitor
TAPI-1	A disintegrin and metalloproteinase inhibitor (ADAM)
U73122	Phospholipase C inhibitor
A23187	Calcium ionophore PKC-activating agent
TMB-8	Intracellular calcium antagonist
R59022	A diacylglycerol kinase inhibitor
PDTC	An inhibitor of NF-kB


TAPI-1; Tumor necrosis factor-alpha (TNF-α) protease inhibitor I, TMB-8; 3,4,5-trimethoxybenzoic acid 8-(diethylamino) octyl ester and PDTC; Pyrrolidinedithiocarbamate.

**Table 2:** Isoform-specific protein kinase C (PKC) inhibitors


Reagent	PKC isoform						Reference no.

Ro-318425	PKCα	PKCβI	PKCβII	PKCγ			11-13
Go 6976	PKCα	PKCβI					14, 15
Go 6983	PKCα	PKCβI		PKCγ	PKCδ		16, 17
CGP41251	PKCα	PKCβI	PKCβII	PKCγ	PKCδ		18, 19
PKC θ/δ inhibitor					PKCδ	PKCθ	20, 21


Xestospongin C, which antagonizes the calciumreleasing action of IP3 at the receptor level, was obtained from Sigma-Aldrich, USA. Each reagent solution was negative for endotoxin according to the Endospecy test (22, 23).

### Isolation of monocytes from peripheral blood mononuclear cells (PBMCs)

Lymphocyte medium for thawing (BBLYMPH1) was obtained from Zen-Bio, Inc. (Research Triangle Park, NC). PBMCs were isolated as described previously (24). Briefly, heparinized blood samples were obtained from nonsmoking healthy volunteers and were diluted 1:1 with pyrogen-free saline. PBMCs were isolated immediately after collection using Lymphoprep gradients (Axis-Shield PoC As, Norway). Then, cells were suspended with BBLYMPH1 and incubated for 3 hours. For monocyte isolation by plastic adherence, 1×10^6^ cells per well were distributed into 12-well plates (Corning Inc. Costar, USA) and allowed to adhere in a 5% CO_2_ incubator at 37˚C for 2 hours. Monocytes were further enriched by virtue of their attachment to a culture plate for 2 hours and washed 3 times with warm phosphate-buffered saline (PBS, Invitrogen, USA) to remove nonadherent cells. Then, monocytes were cultured in complete medium consisting of RPMI 1640 supplemented with 10% heat-inactivated fetal calf serum (FCS), and 10 μg/mL gentamicin (Invitrogen, USA) at 37˚C in 5% CO_2_ humidified air. The adherent monocytes were recovered with a cell scraper. The purity of monocytes was evaluated by fluorescent staining with CD14-phycoerythrin (PE) mouse anti-human monoclonal antibody (Life technologies, USA) and fluorescence-activated cell sorting (FACS) analysis. The recovery of monocytes was also evaluated by trypan blue staining and counted using a microscope (Zeiss, Germany). Only isolated CD14+ monocytes of >85% purity were used for each experiment. After monocytes were resuspended in RPMI-1640 medium supplemented with 25 mM HEPES (Sigma-Aldrich, St. Louis, MO), 100 mmol/L L-glutamine (Invitrogen, Carlsbad), 100 U/mL penicillin100 μg/mL streptomycin (Biowest LLC, USA), and 10% FCS, the cells were stimulated with HNE for 6 hours.

### Extraction of RNA and reverse transcription polymerase chain reaction (RT-PCR)

Extraction was done with 500 μL of TRIzol™ reagent (Invitrogen, France), and total RNA was isolated and precipitated according to the manufacturer’s instructions. Then 1 μg of total RNA was subjected to reverse transcription using random heptamer primers with Moloney murine leukemia virus (Invitrogen, USA). Next, 1 μl of reverse-transcribed RNA was amplified by polymerase chain reaction (PCR) on an ABI PRISM 7000 thermal cycler (Applied Biosystems, USA) using the Taqman™ Master Mix Kit. Quantification of target mRNA was performed by comparing the number of cycles required to reach the reference and target threshold values (25). Monocytes were incubated with HNE (0 or 5 μg/mL) for 6 hours, after which *IL-10* mRNA expression was analysed by RT-PCR. The primer sequences used were as follows:

IL-10 F: 5´-ATGCCCCAAGCTGAGAACCAAGAC-3´
  R: 5´-TCTCAAGGGGCTGGGTCAGCTATCCCA-3´
β-actin F: 5´-GTGGGGCGCCCCAGGCACCA-3´
  R: 5´-CTCCTTAATGTCACGCACGATTTC-3´

The PCR conditions were as follows: for IL-10, 35 cycles (94˚C for 60 seconds, 60˚C for 30 seconds, and 72˚C for 60 seconds) and for β-actin, 40 cycles (94˚C for 30 seconds, 60˚C for 30 seconds, and 72˚C for 30 seconds) were used. The PCR products were analyzed on agarose gels.

### Enzyme-linked immunosorbent assay (ELISA) for Interleukin-10

After monocytes were stimulated with HNE for 6 hours, the level of IL-10 protein in the supernatant was measured by enzyme-linked immunosorbent assay (ELISA) with an anti-IL-10 monoclonal antibody (Abcam Inc., USA).

### Protein kinase C activity assay

PKC kinase activity assay kit was obtained from Abcam Inc. (USA). This kit is based on a solid phase ELISA that utilizes a specific synthetic peptide as a substrate for PKC and a polyclonal antibody that recognizes the phosphorylated form of the substrate. Monocytes were incubated for 6 hours with or without HNE (5 μg/mL). Then, cells were lysed in 1 mL of lysis buffer, and 30 μL were tested for PKC activity.

### Statistical analysis

Data are expressed as the mean ± SD. Analysis of variance and the t test of independent means were used to determine differences between multiple groups and differences between two groups, respectively. When the F ratio was significant, mean values were compared using a post hoc Bonferroni’s test. A P value<0.05 was considered to indicate a significant difference in all analyses.

## Results

**Fig.1:**
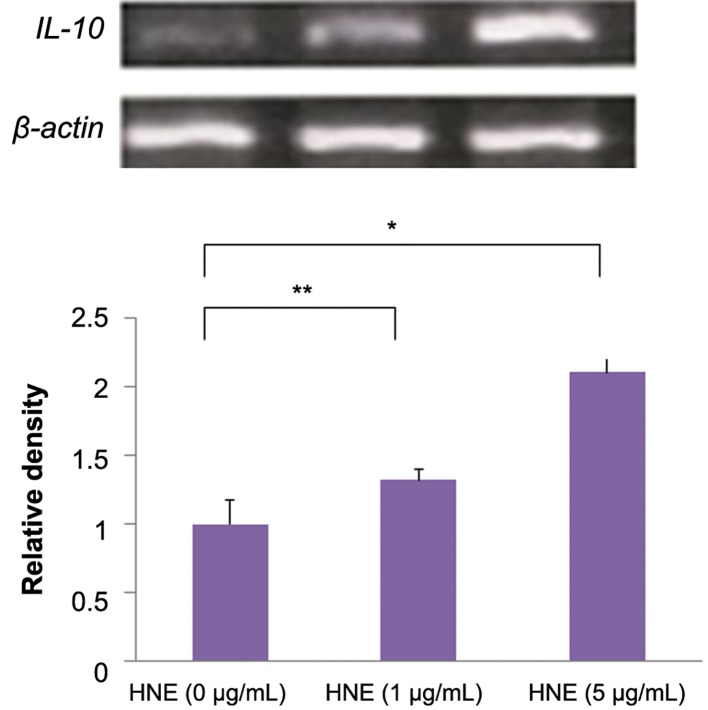
RT-PCR detection of *IL-10* mRNA in monocytes stimulated with HNE. When monocytes were stimulated with HNE (0, 1, or 5 μg/mL), *IL-10* mRNA expression increased in a dose-dependent manner. The relative density of the bands was normalized to β-actin. Data were obtained from three individuals in each group and represent the mean ± SD. *; P<0.01, **; P<0.05, RT-PCR; Reverse transcription polymerase chain reaction, IL-10; Interleukin-10 and HNE; Human neutrophil elastase.

**Fig.2:**
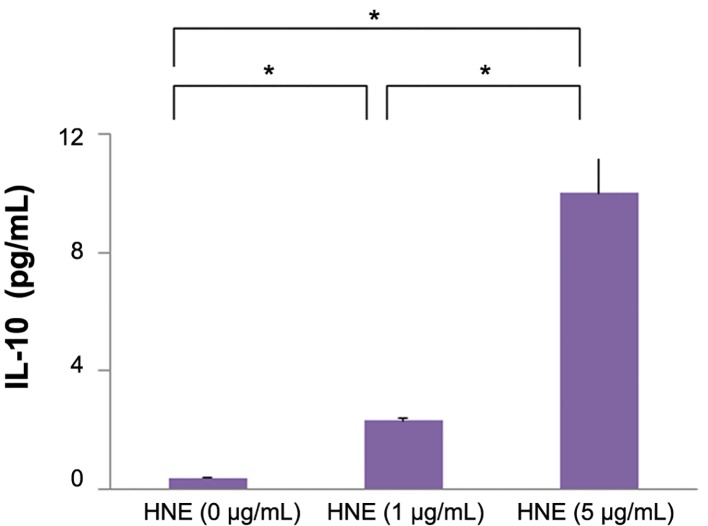
Measurement of IL-10 protein levels by ELISA. IL-10 levels were significantly increased in culture supernatants of monocytes stimulated with HNE (1 or 5 μg/mL) compared with the control (0 μg/mL HNE). Data were obtained from three individuals and represent the mean ± SD. *; P<0.01, IL-10; Interleukin-10, HNE; Human neutrophil elastase and ELISA; Enzyme-linked immunosorbent assay.

**Fig.3:**
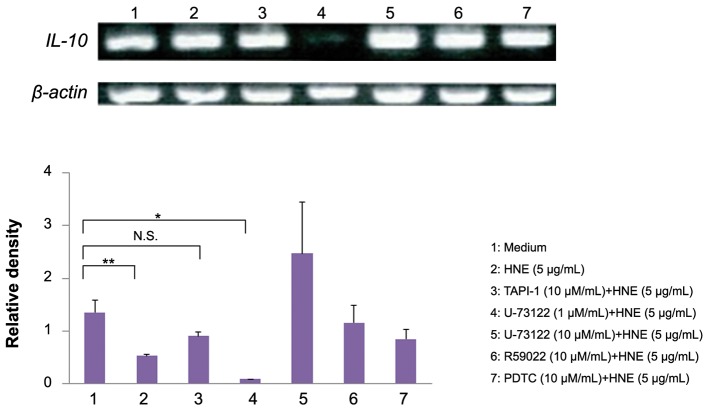
Effect of TAPI-1, U73122, R59022, and PDTC on *IL-10* mRNA expression. U73122 blunted the increase of *IL-10* mRNA in HNE-stimulated monocytess. The relative density of the bands was normalized to β-actin. Data were obtained from three individuals in each group and represent the mean ± SD. *; P<0.01, **; P<0.05, N.S.; Not significant, TAPI-1; Tumor necrosis factor-alpha (TNF-α) protease inhibitor I, PDTC; Pyrrolidinedithiocarbamate, IL-10; *Interleukin-10* and HNE; Human neutrophil elastase.

**Fig.4:**
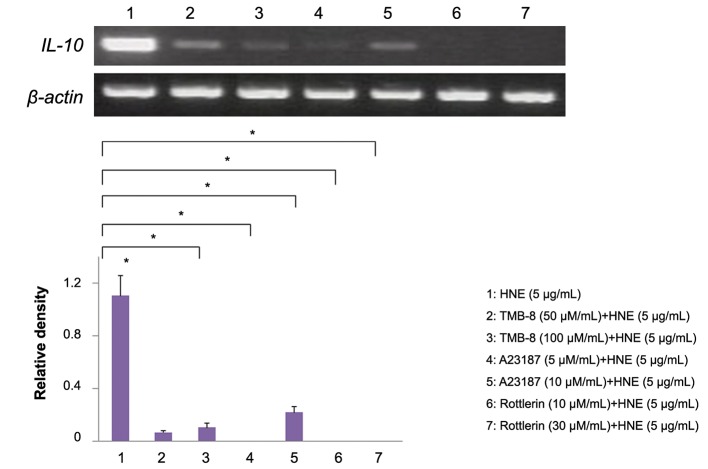
Effect of TMB-8, A23187, and Rottlerin on *IL-10* mRNA expression. TMB-8 partially blocked the increase of *IL-10* mRNA in HNEstimulated monocytes and Rottlerin abolished it. The relative density of the bands was normalized to β-actin. Data were obtained from three individuals in each group and represent the mean ± SD. *; P<0.01, TMB-8; 3,4,5-trimethoxybenzoic acid 8-(diethylamino)octyl ester, *IL-10*; Interleukin-10 and HNE; Human neutrophil elastase.

**Fig.5:**
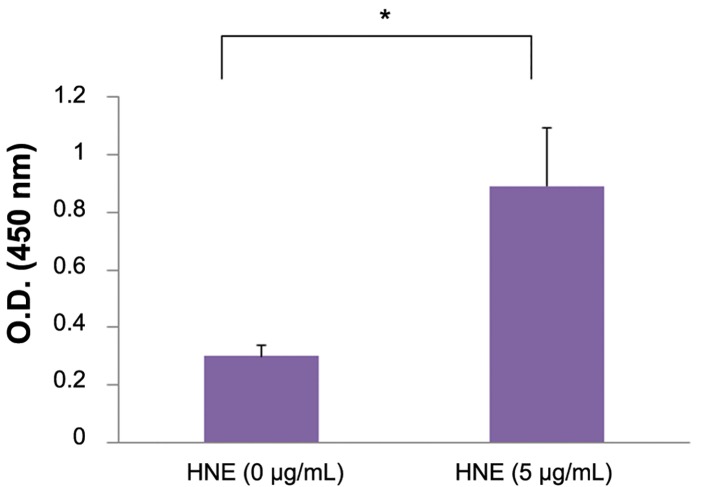
PKC activity assay. Monocytes were incubated for 6 hours with or without HNE (5 μg/mL) and then PKC activity was determined. PKC activity in lysates obtained from monocytes stimulated with HNE was significantly higher than untreated control cells. Data were obtained from three individuals in each group and represent the mean ± SD. *; P<0.01, PKC; Protein kinase C, HNE; Human neutrophil elastase and O.D.; Optical density.

To examine IL-10 response in PBMC, we detect IL-10 expression by semi-quantitative RT-PCR with HNE treatment for 6 hours at concentrations of 0, 1, and 5 μg/mL. The relative IL-10 expression is around 2 times higher with HNE treatment at 5 μg/mL than control (0 μg/mL). Consistent with the data revealing PBMCs increase IL-10 expression after HNE treatment (Fig .1), secretion IL-10 protein are also increased around 10 times in supernatants after HNE treatment by ElISA (Fig .2). The U73122 (phospholipase C inhibitor) significantly reduced the response of *IL-10* mRNA expression to stimulation with HNE. In contrast, R59022 (a diacylglycerol kinase inhibitor) had no effect on IL-10 mRNA levels. Similarly, neither a TNF-α converting enzyme (TACE) inhibitor, TAPI-1, nor an inhibitor of nuclear factor-kappa B (NF-kB), PDTC, reduced *IL-10* mRNA expression by HNE-stimulated monocytes (Fig .3). However, the calcium chelator, like TMB-8, completely inhibited the response of *IL-10* mRNA to HNE, although calcium ionophore A18237 (a PKC-activating agent) did not augment *IL-10* mRNA expression. Interestingly, the PKC inhibitor Rottlerin blunted the increase of IL-10 mRNA expression after stimulation of monocytes with HNE (Fig .4). Monocytes were incubated for 6 hours with or without HNE (5 μg/mL) and then PKC activity was determined. PKC activity in lysates obtained from monocytes stimulated with HNE was significantly higher than untreated control cells (Fig .5).

**Fig.6:**
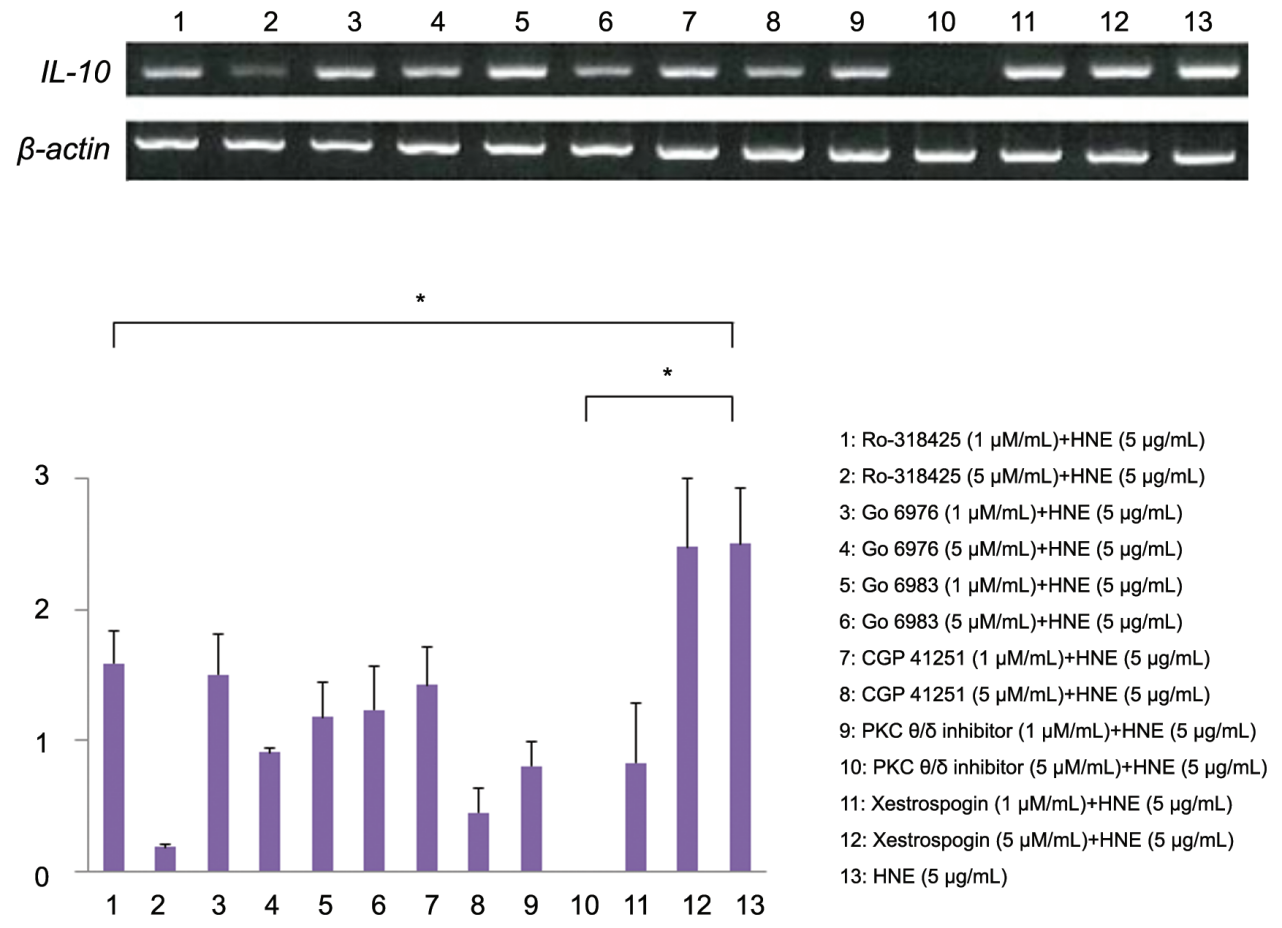
Effect of various PKC isoform inhibitors on *IL-10* mRNA expression by monocytes after HNE stimulation. Ro-318425 partially inhibited the increase of *IL-10* mRNA expression in response to HNE, while the PKC theta/delta inhibitor completely abolished it. The relative density of the bands was normalized to β-actin. Data were obtained from three individuals in each group and represent the mean ± SD. *; P<0.01, PKC; Protein kinase C, *IL-10*; Interleukin-10 and HNE; Human neutrophil elastase.

Next, the effect of various PKC isoform inhibitors on *IL-10* mRNA expression was examined by RT-PCR. Ro-318425 (1 μM/mL) partially inhibited the increase of *IL-10* mRNA expression in monocytes exposed to HNE and more effectively inhibited the response of *IL-10* mRNA at a higher concentration (5 μM/mL). Go 6976 (1 μM/mL) had no influence on the increase of *IL-10* mRNA in monocytes exposed to HNE, but partially inhibited the response of *IL-10* mRNA at a higher concentration (5 μM/mL). Similarly, Go 6983 had no effect at a concentration of 1 μM/mL, but partially inhibited *IL-10* mRNA expression at a concentration of 5 μM/mL. Interestingly, addition of a PKC theta/delta inhibitor (5 μM/mL) completely abolished IL-10 expression (Fig .6).

## Discussion

The present study demonstrated that the phospholipase C inhibitor, U73122, blunted the upregulation of *IL-10* mRNA expression in response to stimulation of monocytes with HNE. U73122 is reported to be as specific inhibitor of G-proteinmediated phospholipase C activation (26). In addition, pre-incubation with TMB-8, a PKC inhibitor that is also an intracellular Ca^2+^ antagonist (27, 28), was more effective at inhibiting the response of *IL-10* mRNA to HNE stimulation.

These findings suggest that HNE upregulates IL- 10 expression in PBMCs by promoting intracellular Ca^2+^ influx and activating the phospholipase C signaling pathway.

Xestospongin C antagonizes the calcium-releasing action of IP_3_ at the receptor level. Inositol phosphates are important signal transduction messengers that act via IP_3_ receptors to promote the mobilization of Ca^2+^ from intracellular stores. Xestospongin C blocks the increase of intracellular calcium and also inhibits the Ca^2+^ ATPase pump in the sarcoplasmic reticulum (29). In the present study, xestospongin C did not affect the increase of *IL-10* mRNA expression in response to HNE stimulation. The protease-activated receptor (PAR) family of G protein-coupled receptors is activated by a unique mechanism that involves proteolytic unmasking of an N-terminal self-activating tethered ligand. Proteinases can either activate PAR signaling by unmasking the tethered ligand sequence or disarm the receptor for subsequent enzyme activation by cleavage downstream from this sequence (30).

Tumor necrosis factor-alpha converting enzyme (TACE) cleaves TNF at the Ala-76–Val-77 site and TACE expression has been detected on alveolar macrophages by flow cytometry. It is known that activation of the epidermal growth factor receptor and its downstream signaling cascade are involved in the production of mucin. TACE cleaves pro-transforming growth factor-α in airway epithelial cells to release its mature soluble form, which subsequently binds to and activates the epidermal growth factor receptor. Shao and Nadel (31) previously demonstrated that HNE induces MUC5AC mucin expression in human airway epithelial cells via a cascade that includes PKC, oxygen radicals, and TACE. However, TAPI-1 did not affect *IL-10* mRNA expression by monocytes in the present study. Instead, the present findings indicated that *IL-10* production by monocytes involves a Rottlerin-sensitive pathway. Rottlerin has been employed as a selective inhibitor of protein kinase C delta in several studies (12, 32). In contrast, Soltoff (33) reported that Rottlerin did not block PKCdelta activity *in vitro*, although it blocked several other kinase and non-kinase proteins and strongly activated multiple Ca^2+^-sensitive K^+^ channels. In the present study, we employed a molecular approach to characterize the role of each PKC isoform in the regulation of HNE-induced *IL-10* expression by monocytes. Because the PKC isozyme family is clearly divided into three subgroups (conventional, novel, and atypical), the role of PKC isoforms from each subgroup in *IL-10* production was investigated. The broad-spectrum PKC inhibitor bisindolylmaleimide Ro-318425 (which inhibits conventional PKC isoforms) partially blocked upregulation of *IL-10* mRNA expression by HNE in a concentration-dependent manner. However, the Ca^2+^-dependent PKC inhibitor Go 6976 (another inhibitor of conventional PKC isoforms) had less effect on the response of *IL-10* mRNA to HNE. Similarly, the broad-spectrum PKC inhibitor Go 6983 (a PKCβII inhibitor) partially blocked the increase of *IL-10* mRNA expression induced by HNE. Interestingly, a PKC theta/delta inhibitor (which inhibits novel PKC isoforms) strongly suppressed IL- 10 mRNA expression after HNE stimulation. These findings suggest that HNE upregulates *IL-10* expression in monocytes by promoting intracellular Ca^2+^ influx, activating phospholipase C, and preferentially activating the novel PKC signaling pathway over the conventional pathway.

## Conclusion

Monocytes produce anti-inflammatory IL-10 after stimulation with HNE. IL-10 production involves intracellular Ca^2+^ and activation of the phospholipase C pathway. IL-10 production also depends on activation of the novel PKC theta/delta, rather than conventional PKC isoforms.
